# Annexin, a Protein for All Seasons: From Calcium Dependent Membrane Metabolism to RNA Recognition

**DOI:** 10.1002/bies.70019

**Published:** 2025-05-12

**Authors:** Anni Vedeler, Gian Gaetano Tartaglia, Annalisa Pastore

**Affiliations:** ^1^ Neurotargeting Group Department of Biomedicine University of Bergen Bergen Norway; ^2^ Center for Human Technologies Istituto Italiano di Tecnologia Genova Italy; ^3^ Elettra Sincrotrone Trieste Basovizza Italy; ^4^ The Wohl Institute King's College London London UK

**Keywords:** amyotrophic lateral sclerosis, annexins, calcium‐binding proteins, function, intrinsically unstructured regions, RNA, phospholipids, structure

## Abstract

Annexins are a protein family well known to bind to phospholipids in a calcium‐dependent way. They are involved in several different crucial cellular processes such as cell division, calcium signaling, membrane repair, vesicle trafficking, and apoptosis. Although RNA binding for some members of the family was reported long ago, it was only recently that it was shown that a common feature of the family is also the ability to bind RNA, a discovery that has added significantly to our perception of the cellular role of these proteins. In the present review, we discuss the properties of annexins under an updated light and the current knowledge on the RNA binding properties of annexins. We then focus specifically on annexin A11, because this is a less characterized member of the family but, at the same time, a potentially important component of the mRNA transport machinery in neurons. We hope to offer to the reader a more complete picture of the annexins’ binding properties and new tools to evaluate the multifaceted functions of this important protein family.

## Graphical abstract

Annexins are an important protein family traditionally well known to bind to phospholipids and to interact with various proteins in a calcium dependent way. More recently, it has been established that a feature common to the family is also to bind to RNA having a role in translation. In bies70019 article, Vedeler et al. discuss the properties of annexins under the current knowledge of their RNA binding properties. They then focus specifically on the less characterized member of the family annexin A11, that is, at the same time, a potential essential component of the mRNA transport machinery in neurons.

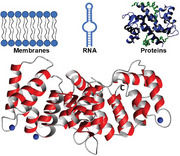

## 1 Introduction

Annexins are a superfamily of proteins that were originally identified in the late 70s/early 80s related to their ability to bind to phospholipids, particularly phosphatidylserine, in a calcium‐dependent manner. The name “annexin” was coined from the Latin word “annectere” for the ability to connect together something, as is the case for annexins to the cellular membranes or liposomes [1]. The interaction often leads to changes in membrane structure and function. Annexins are found in several different tissues and cell types in higher and lower eukaryotes, including mammals, birds, fish, amphibians, *Drosophila*, *Dictyostelium*, and plants [2]. Twelve distinct annexins (AnxA1‐AnxA11 and AnxA13) are known in humans, of which AnxA7, AnxA11, and AnxA13 are considered the oldest members of the group: the nine descendent annexins (AnxA1, AnxA2, AnxA3, AnxA4, AnxA5, AnxA6, AnxA8, AnxA9, and AnxA10) are assumed to originate from the common ancestor AnxA13 [3, 4, 5]. Among these annexins, only AnxA10 is unable to bind to liposomes containing negatively charged phospholipids at physiological Ca^2+^ concentrations but is found in paraspeckles [6]. Paraspeckles are structures in the nucleus suggested to act as hubs regulating genome organization, and as a preassembly site for mRNAs [7].

According to the first definition, to classify a protein as an annexin, it was necessary and sufficient to observe lipid binding in a calcium‐dependent way and that the protein contains a well‐defined sequence motif [1]. However, after a long debate, a new function has eventually been added to the annexins’ portfolio: that of RNA‐binding, indicating an involvement of these proteins in RNA‐related processes [8]. This function well agrees and substantiates previous reports that have linked annexins to RNA metabolism. Positive and polar residues in helices C‐D in the fourth annexin repeat of AnxA2 have been shown to bind to *cis*‐acting elements in the 3'UTRs of various mRNAs and to contribute to posttranscriptional regulation of the expression of specific genes (reviewed in ref. [9]). More recently, a function as a tethering element between lysosomes and RNA granules was attributed to AnxA11, thus facilitating the hitchhiking of granules on the membranous organelles [10].

In the present review, we scrutinize the annexin literature and discuss the binding properties of the whole family, comparing structural and functional features and paying specific attention to their mRNA binding properties, an aspect that has attracted, on the whole, still too little attention. We then focus on AnxA11, one of the progenitors of the annexin family, to predict the mode of interaction of this protein with specific protein partners. We hope that this review will provide a new and more thorough perspective of the complex cellular role of annexins in RNA metabolism.

## 2 Structural Features of the Family

**FIGURE 1:**
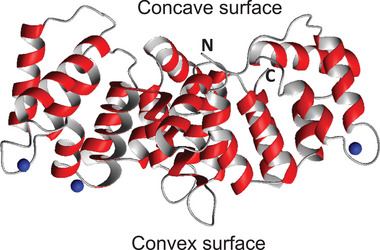
Ribbon structure of the conserved core domain of ANXA11 with calcium ions bound (6tu2). The convex and concave surfaces are indicated, together with the N‐ and C‐termini.

**FIGURE 2:**
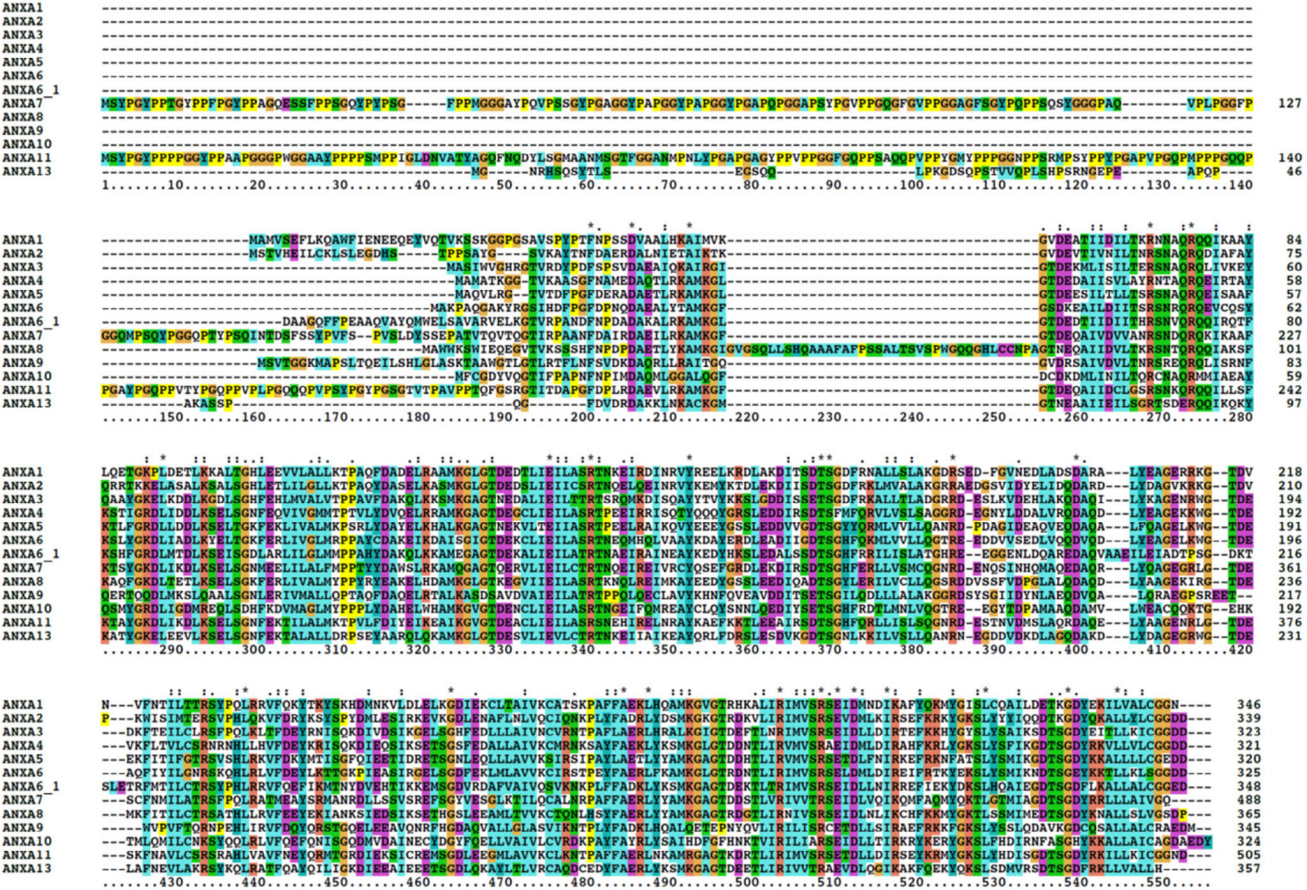
Multiple alignment of the human annexin sequences. It shows the high conservation of the C‐terminal core domain and the low conservation of the N‐terminus. AnxA6, which is the only annexin containing two copies of the four annexin motifs, is identified as AnxA6 and AnxA6_1 for the N‐ and C‐terminal halves, respectively. The alignment was produced with clustalX (www.clustal.org/clustalx).

**FIGURE 3:**
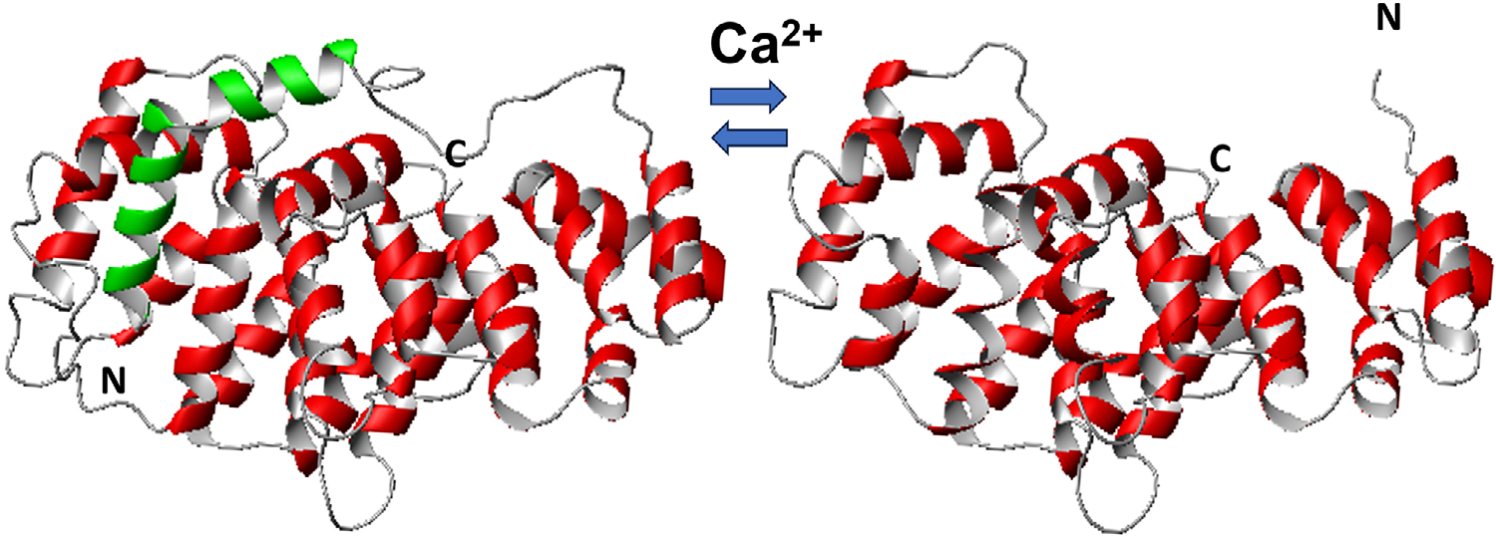
Comparison of the ribbon structures of Ca^2+^‐free (left, 1HM6) and Ca^2+^‐loaded (right, 1CMX) ANXA1. The conserved core domain is shown in red. In the Ca^2+^‐free form the (short) N‐terminus (in green) folds back and inserts into the globular domain. In the Ca^2+^‐bound form a conformational rearrangement of the helices causes the expulsion of the N‐terminus, which becomes very flexible and unstructured and is therefore not detected. The N‐ and C‐termini are indicated.

The first structure of an annexin was solved by Huber's group in 1990 for AnxA5 [11]. The structure is formed by an evolutionarily conserved core domain that contains four (except for AnxA6, which has eight) repeats of an α‐helical motif of approx. seventy residues, each of which comprises five helices. This fold was already identified and structurally predicted based on homology in an earlier paper [1]. The annexin repeats were identified as a calcium‐binding motif distinct from EF‐hands and C‐motifs. The whole conserved C‐terminus of annexins (annexin core domain) forms a compact, slightly curved disc with its convex surface harboring the calcium and membrane‐binding sites, whereas the concave side points away from the membrane and is available for other types of interaction/regulation (Figure 1). The annexin core domain is preceded by an N‐terminus that is highly variable both in sequence and length, ranging from less than 20 residues to around 200 (Figure 2 and Table 1). There is no homology between the different N‐termini, and thus this region is supposed to provide functional specificity. Calcium seems, for instance, to have a regulatory role depending on the subfamily and on the N‐terminus sequence. Typically, the AB‐loop and the DE‐loop harbor the Ca^2+^‐binding sites in the annexins [12, 13, 14]. AnxA1, for instance, has an N‐terminus of approx. thirty residues that form a helix in the absence of calcium, which inserts between the core helices by displacing two of them [15]. AnxA1 has a high affinity for Ca^2+^ and can bind up to eight Ca^2+^ ions (predicted by similarity). When calcium binds, the core domain undergoes a conformational rearrangement that results in the expulsion of the N‐terminal helix, which becomes exposed to the environment and proficient to form other interactions (Figure 3) [15]. A similar mechanism was hypothesized for AnxA2 [16]. Molecular motion analyses based on structural coordinates (1W7B and 1XJL) of AnxA2 have also shown that the largest changes in the backbone conformation occur at Gly100, Thr133, Gly157, and Gly163. In addition, Ca^2+^ induces changes in the backbone dihedral torsion angles at Gly312 in the CD loop and thus also in the orientation of helix D relative to helix C (for details, see ref. [17]). Regarding binding to mRNA, it has been suggested that Ca^2+^‐binding increases the accessibility of the RNA‐binding site by changing the orientation of the very flexible region of the N‐terminal end of AnxA2 [18]. AnxA7 and AnxA11 have much longer N‐termini of 201 and 216 residues, respectively, with low complexity sequences rich in prolines and glycines. Little is known about their regulation. The annexin proteins have different calcium affinities in the µM range [19, 20, 21], which may regulate their response to calcium signaling. Most annexins exist in several isoforms due to alternative splicing. This primarily results in N‐ or C‐terminal truncated protein variants, as well as several internally truncated versions. It is likely that some of these different isoforms bind to different ligands, potentially altering the ligand specific regulation of the annexin functions. While all human annexins have isoforms, rat AnxA1, AnxA3, AnxA4, AnxA9, AnxA10, and AnxA13 appear to only exist in one form. Interestingly, introducing only two extra amino acids (Ser‐Gln) in the N‐terminus of rat AnxA2 introduces a potentially new phosphorylation site   [22].

**TABLE 1:** Summary of the properties of human annexins.

Annexin	N‐term length^1^	Total length
AnxA1	58	346
AnxA2	49	339
AnxA3	34	323
AnxA4	32	321
AnxA5	31	320
AnxA6	36	673
AnxA7	201	488
AnxA8	75	365
AnxA9	57	345
AnxA10	33	324
AnxA11	216	505
AnxA13	71	357

*Note*: All of them have in the C‐terminal core domain four annexin repeats, except for AnxA6, which has eight. The N‐terminal four repeats of AnxA6 are most similar to AnxA10, and the C‐terminal four repeats are most similar to AnxA5 [29].

^1^The N‐terminus is defined up to the detection of the first annexin repeat as defined in SMART.

Posttranslational modifications (PTMs) of proteins represent an additional level of regulation for protein function. Annexins undergo various modifications. These modifications have been most extensively studied for AnxA2 and include acetylation [23], phosphorylation [24], ubiquitination [25], and SUMOylation [26]. PTMs are involved in the regulation of binding to ligands; examples are phosphorylation of Ser5 in AnxA1 and of Ser12 (counting the first Met as amino acid 1) in AnxA2, which prevents the interaction with S100A11 and S100A10, respectively [27, 28]. It is not surprising that the functions of annexins are regulated at various levels since these proteins are truly multifunctional proteins destined to regulate and coordinate several cellular processes in response to different signals. Annexins are Ca^2^⁺‐binding proteins that play diverse roles in cellular functions both intracellularly and extracellularly. These functions include signal transduction, cell proliferation, binding and trafficking of membranes and mRNA, interactions between membranes and the cytoskeleton, as well as wound healing [9, 19, 21, 29–31]. Thus, annexins are essential cellular proteins, and it is likely that there are redundancy/compensatory effects among the annexin family members. In this connection, it is interesting to note that when AnxA2 was knocked down in PC12 cells, the level of AnxA7 appeared to increase several folds [32]. Both AnxA2 and AnxA7 bind mRNAs, Ca^2+^, and acidic phospholipids [8, 20, 21, 31]. Only a limited number of mRNAs bound to AnxA7 have been identified thus far [8]. Both annexins are associated with the c‐*myc* 3´UTR, while only AnxA2 binds to the c‐*myc* 5´UTR [8]. Interestingly, higher expression of AnxA7 is linked to a better prognosis in breast cancer patients [33], whereas higher expression of AnxA2 is correlated with worse outcomes [34]. Taken together, these findings suggest that there may be some redundancy, particularly concerning the RNA‐binding roles of the two annexins.

### 2.1 RNA‐Binding of Annexins

The first indications that AnxA2 binds RNA were reported in 1983 by Arrigo and colleagues. The authors discovered a small subpopulation of an unidentified protein associated with small RNAs in mRNP complexes [35] that was later on identified as AnxA2 [36]. The small RNAs identified may have been either degraded mRNA or small regulatory RNAs since not only mRNAs but also regulatory RNAs have been found to associate with AnxA2 [37]. Vedeler and coworkers were the first to demonstrate that AnxA2 acts as an mRNA‐binding protein associated with a specific subpopulation of messenger ribonucleoprotein (mRNP) complexes linked to the cytoskeleton [9, 17, 36, 38, 39]. The c‐*myc* mRNA, a member of this subpopulation of mRNAs, was subsequently found to bind to AnxA2 [40]. AnxA2 in its monomeric form was also found to bind directly distinct RNA sequences within the 3´UTRs of *anx*A2 and c‐*myc* mRNAs containing higher order structures with a five nucleotide consensus sequence 5´‐AA(C/G)(A/U)G‐3’ [38, 39]. These are mRNAs translated mainly on cytoskeleton‐bound polysomes [9, 36]. However, AnxA2 binds to NMDA R1 mRNA as a monomer, and NMDA R1 mRNA is translated only on membrane‐bound polysomes on the rough endoplasmic reticulum [41]. AnxA2 regulates c‐Myc expression by binding to the 5′UTR of its mRNA in a Ca^2+^‐dependent manner at the two pseudoknots of the internal ribosome entry site (IRES) [18]. AnxA1, AnxA10, and AnxA11 are other examples of what we now consider bona fide RNA‐binding annexins [6, 10, 42]. More recently, the Vedeler's group demonstrated, using *anx*A2 and c‐*myc* 3´ and 5’ UTRs as baits, that most annexins bind to RNA and showed the presence of selected annexins in mRNP complexes derived from the neuroendocrine rat PC12 cells [8]. This evidence suggests that RNA‐binding is an ancient feature common to the whole annexin protein family. Using biolayer interferometry (BLI) to study in more detail the apparent dissociation constant K_D_ of the interactions between selected annexins and the c‐*myc* or *anx*A2 3′UTR, both selectivity and specificity of the interactions were detected. AnxA2, AnxA13, and the core structures of AnxA7 and AnxA11 bound the c‐*myc* and *anx*A2 3′‐UTRs with K_D_s in the nM range (∼75–250 nM), while the core structure of AnxA11 has a much lower affinity for *anx*A2 3′UTR (∼2 µM) than c‐*myc* 3´UTR. Only AnxA2 binds to the 5′UTR of c‐*myc* mRNA, most likely related to the regulation of translation. AnxA4 did not bind to the two specific 3′UTRs, but to a pool of total mRNAs (for details, see ref. [8]). Through the Vedeler's work [8], it was also clear that annexins do not simply bind to RNA generically and nonspecifically, but seem to be associated with complexes made of mRNPs, which include proteins that are bound directly or indirectly to mRNAs while they are being synthesized, spliced, exported, transported, and translated in the cytoplasm [43, 44]. This implies a role that well agrees with the combined calcium‐regulated RNA‐ and lipid‐binding properties observed for the annexin family, in coordinating long‐distance transport of membrane vesicles and mRNAs, regulated by Ca^2+^ [10]. This is an essential function that plays a central role in the traffic of molecules between different membrane‐enclosed compartments of the secretory pathway. In support of this hypothesis, AnxA2 was demonstrated to colocalize with the P‐body marker GW182 [45], while AnxA1, AnxA6, AnxA7, and AnxA11 have been identified in stress granules. These are spatially, compositionally, and functionally linked complexes of stalled translationally inactive mRNAs [46, 47]. AnxA1, AnxA2, AnxA4, AnxA5, AnxA6, AnxA7, AnxA10, AnxA11, and AnxA13 were all detected in non‐polysomal mRNP complexes [8]. Thus, like in membrane repair [48, 49], several annexins function together in mRNP complexes, possibly each having preferences for specific mRNAs. P‐bodies and/or stress granules regulate the temporary translational repression and decay of mRNAs. These RNP condensates are dynamic complexes that share some of the same proteins and act upon cellular signaling.

### 2.2 A Role of Annexins in Translation

**FIGURE 4:**
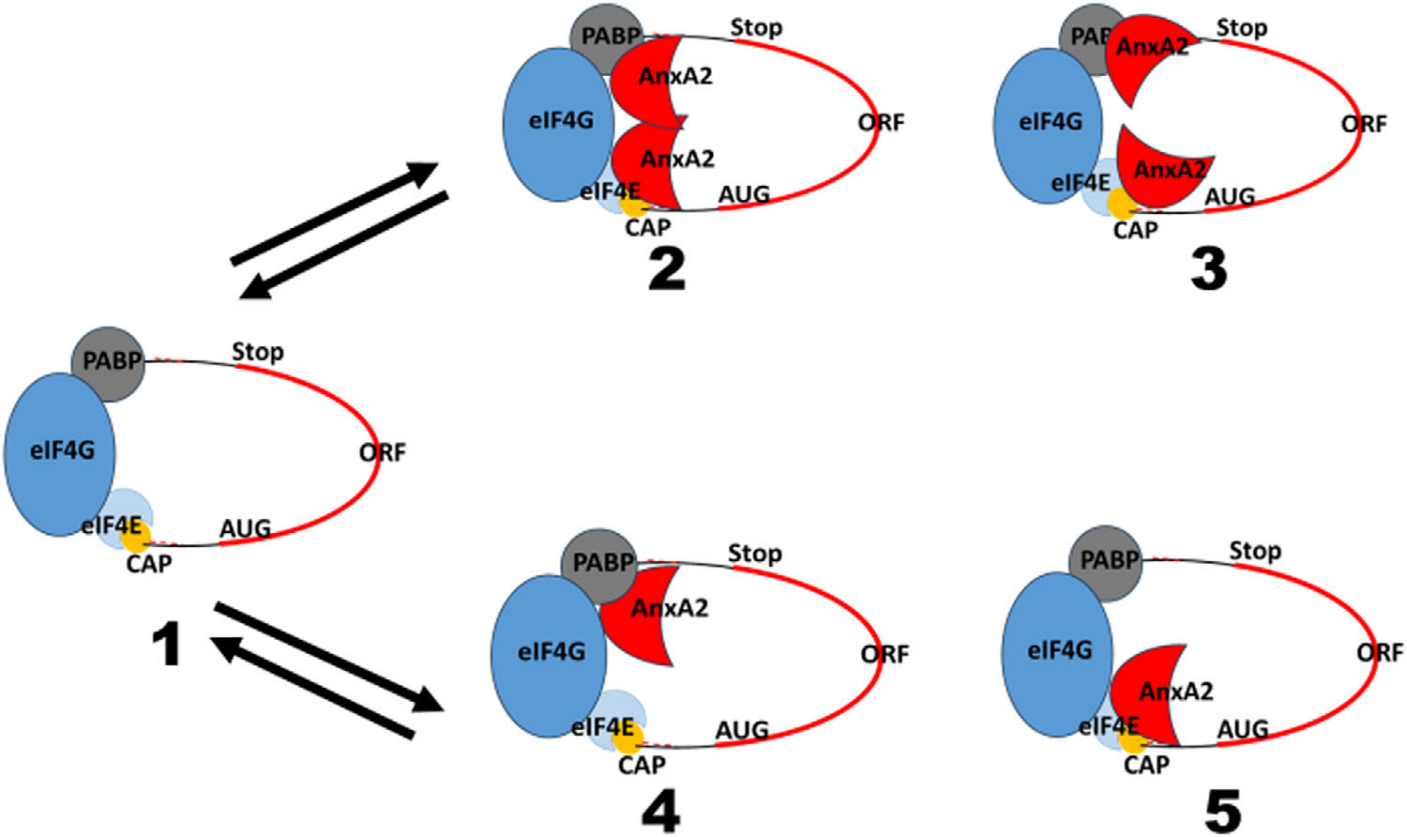
Proposed models for the association of AnxA2 with the eIF4F initiation complex and/or PABP and its effect on the translation of *anx*A2 mRNA. Schematic model of an mRNA ready to form the preinitiation complex (1). AnxA2 is present in translationally inactive mRNP complexes and likely inhibits translation by binding to the eIF4F initiation complex and/or PABP (2–4). AnxA2 could be bound as a dimer (2–3) or a monomer (4–5). At present it is unknown which form is involved in the putative tethering to vesicles during mRNA transport.

Little is known about the regulation of annexins and mRNA binding, with most knowledge being related to AnxA2. Calcium has been identified as an important factor for binding, presumably to unmask the RNA‐binding site(s) by inducing a conformational transition [17, 40, 50]. It was also observed that Ser25 phosphorylation, in combination with ubiquitination and SUMOylation, appears to target AnxA2 to perinuclear inactive RNA granules [45]. AnxA2 binds the c‐*myc* IRES, resulting in a dose‐dependent inhibition of translation [18]. Moreover, it was shown that AnxA2 binds eIF4E (possibly eIF4G) and PABP1 in an RNA‐independent manner [51]. By binding to PABP1 and subunits of the initiation complex eIF4F, AnxA2 prevents the formation of the full eIF4F complex [51] (Figure 4). This may explain the presence of AnxA2 in translationally inactive mRNP complexes and its involvement in the regulation of translation initiation.

The initiation of translation is highly regulated, and the rate limiting step of translation. This is important as translation requires a high amount of energy. Cap‐dependent translation relies on the binding of the eukaryotic initiation factor eIF4F complex consisting of eIF4G, and eIF4A as well as eIF4E, which binds directly to the 5′ cap structure of the mRNA before recruitment of the small ribosomal subunit and other initiation factors. The regulation of the binding of eIF4F to mRNA can be exerted by the sequestering of its subunits by binding to ligands. Circularization of mRNA also appears to be important for the initiation of translation. This is mediated by the interaction of the poly(A)‐binding protein (PABP) with eIF4G. This 43S preinitiation complex scans the mRNA until the first 5´‐end located AUG codon is recognized and translation subsequently starts (for details, see refs. [52, 53]).

When cap‐dependent initiation of translation is compromised, the mRNAs containing an IRES can use an alternative mechanism of translation initiation, in which translation does not start at the very 5´‐end of the mRNA but further down‐stream on the mRNA. IRES‐dependent translation relies on the association/dissociation of IRES‐*trans*‐acting factors (ITAFs) for recruitment of the small ribosomal subunit [54]. Four ITAFs, namely GRSF‐1 (G‐rich RNA sequence binding factor 1), YB‐1 (Y‐box binding protein 1), PSF (polypyrimidine tract binding protein‐associated splicing factor), and its binding partner, p54nrb, were found to bind to the c‐*myc* IRES and positively increase IRES‐dependent translation of the c‐*myc* mRNA [55]. AnxA2 also binds to the c‐*myc* IRES but inhibits translation [18]. Thus, it was suggested that AnxA2 could act as a Ca^2+^‐dependent switch between cap‐dependent and IRES‐dependent translation of c‐*myc* mRNA [18], most likely involving specific PTMs. Other interesting interactions are also likely to be discovered since *cis*‐acting sequences for RNA‐binding proteins (RBPs) can be extracted using the BLAST software (https://blast.ncbi.nlm.nih.gov/Blast.cgiI) or algorithms such as catRAPID (http://s.tartaglialab.com/page/catrapid_group) to detect additional putative RNAs regulated by the same RBPs.

Annexins also bind to viral RNA, allowing viruses to utilize the cellular machinery for various functions. This interaction has primarily been studied for AnxA2, with a recent review providing detailed insights into this topic [56]. It was found that AnxA2 is involved in several aspects of the viral life cycle by interacting with viral proteins that facilitate the attachment of virions to receptors, viral gene expression, replication, intracellular trafficking, and virus assembly for both DNA and RNA viruses [56]. These roles align well with AnxA2's RNA‐binding capabilities and its ability to bind to cellular membranes [9, 21]. One example is the interaction between AnxA2 and the human immunodeficiency virus (HIV) Gag protein, which occurs in late endosomes and/or multivesicular bodies (MVBs) [57], suggesting a role for AnxA2 in coordinating vesicle and viral RNA transport. Another example is the high‐affinity complex formed between AnxA2 and hepatitis C virus (HCV) nonstructural protein NS5B, where AnxA2 and NS5B exhibit different preferences for RNA [58]. This suggests that AnxA2 could coordinate the intracellular transport of both cellular mRNAs and viral RNA. Additionally, AnxA2 appears to be involved in the formation of the HCV replication complex located on lipid rafts [59]. Interestingly, following the COVID‐19 pandemic, it was found that AnxA2 binds to a pseudoknot structure of avian infectious bronchitis virus (IBV) RNA—a member of the *Coronaviridae* family that includes SARS‐CoV—reducing the efficiency of ‐1 ribosomal frameshifting. This supports a role for AnxA2 in the synthesis of proteins involved in viral replication [60].

### 2.3 Predicting RNA Partners of Annexins

**FIGURE 5:**
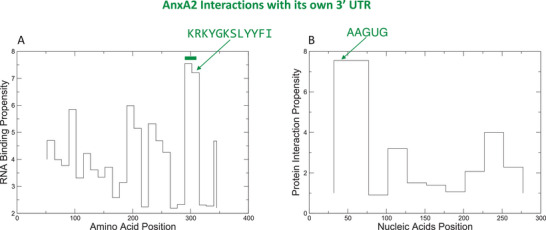
AnxA2 interactions with its own 3' UTR. (A) RNA‐binding ability of different AnxA2 (UniProt Q07936) regions for its own 3' UTR. The region corresponding to the consensus motif is highlighted in green. Note that each position on the x‐axis represents ±25 amino acids. (B) The protein‐binding ability of the A2 transcript (3' UTR) indicates that the upstream region is contacted by AnxA2. In the calculations, the poly‐A tail was omitted due to varying lengths in transcript isoforms. The calculations were performed using catRAPID 2.1 omics.

A recent study of the RNA‐binding properties of annexins [61] demonstrated that catRAPID, an algorithm designed to predict RNA‐protein interactions (http://s.tartaglialab.com/page/catrapid_group) using physico‐chemical properties of RNA and protein sequences [62, 63], is able to correctly predict known binding properties of the annexin family. The possibility of relying on an efficient predicting tool for the prediction of specific RNA partners, as it seems to be the catRAPID suite, has opened new perspectives to fully understand the structural determinants and the specificity of RNA binding. Significant variations were observed among different annexins and their interaction with the 3´ UTR and 5´ UTR of c‐myc. AnxA2 was found to bind more strongly with the 5´ UTR than with the 3´ UTR of c‐*myc*, whereas AnxA7 and AnxA11 showed a preference for the 3´ UTR. AnxA13 exhibits a lower, yet significant, RNA binding ability, while AnxA4 showed a markedly lower affinity compared to AnxA2, AnxA7, and AnxA11. Exploiting the predicting power of catRAPID, the authors also constructed a virtual library of potential mRNA partners for AnxA2. Further analysis of different regions within the AnxA2 sequence indicated varying binding propensities. The C‐terminal core domain, particularly within the fourth annexin repeat encompassing the KKKYG(+DFPL)KSLY motif, exhibited a stronger propensity for interaction (Figure 5A). Notably, AnxA2 binds to its own 3′ UTR by recognizing the AAGUG motif at the 5′ end, consistent with previous findings (Figure 5B) [38, 39]. In contrast, the N‐terminus demonstrated poor binding ability. For AnxA7, a prominent RNA‐binding region was identified in the C‐terminus, with a potential additional interacting region in the middle (amino acids 240–260). Similarly, AnxA11 showed RNA‐binding ability in multiple regions, including both N‐ and C‐termini, with the C‐terminus corresponding to the consensus motif. Additionally, the authors predicted that AnxA7 and AnxA11 have a high propensity for phase separation, in line with reports of the presence of these proteins in stress granules. The phase separation propensity profiles for both AnxA7 and AnxA11 highlighted the N‐terminus as contributing significantly to the formation of large assemblies. This study furthered our understanding of the RNA‐binding properties of annexins and gave us confidence about the possibility of accurately predicting in silico the propensity of a protein to interact with RNA. This possibility could also be helpful for other RBPs.

### 2.4 The Overall Functional Role of Annexins

The study of annexins continues to raise increasing interest, mainly because a better understanding of their roles in cellular processes could also explain their potential implications in various diseases. There is, for instance, a growing consensus that annexins play a role in cancer and that various members of this protein family are heavily involved in tumor cell proliferation, cell cycle regulation, invasive metastasis, apoptosis, and autophagy in several different cancer types and act as mediators of tumorigenesis, proliferation, and metastasis [64, 65, 66]. A recent review has addressed the latest data regarding the biological properties and functions of annexins in various types of cancer, as well as discussed their potential as novel therapeutic targets in cancer treatment [67]. Generally, the involvement of annexins in cancer appears to result not from mutations or deletions in the proteins, but rather from deregulation of their expression levels [67]. As previously discussed for AnxA2, this protein binds to its cognate mRNA, creating a feedback mechanism that may regulate transport and/or the initiation of translation, with outcomes influenced by different signaling [9]. This feedback mechanism may also apply to other annexin family members. Interestingly, annexins are present in exosomes and micro‐vesicles, where they associate with coding and noncoding RNAs [37], thus playing a role in horizontal transfer, for example, between cancer cells and their microenvironment.

Annexins have also been implicated in neurodegeneration, as their mutations and malfunctioning have been associated with amyotrophic lateral sclerosis (ALS), frontotemporal dementia, and Alzheimer's disease [68], having a multifaceted contribution to both neuroprotection and pathology. AnxA1 is, for instance, particularly important for its anti‐inflammatory properties, promoting resolution of inflammation by interacting with formyl peptide receptors (FPRs). In neurodegenerative diseases like Alzheimer's and Parkinson's, dysregulation of inflammation, AnxA1 helps to modulate microglial activation and cytokine production. Neurons are highly dependent on membrane integrity. Annexins (e.g., A2, A6) contribute to membrane repair mechanisms and play a protective role in sealing microlesions. Some annexins (like Annexin A5) can bind to phosphatidylserine on the outer leaflet of apoptotic cells, often used as a marker of apoptosis, but may also be involved in regulating cell death signaling. This is relevant in diseases like ALS and Huntington's, where neuronal death is a central feature.

Annexins are found in a variety of cell types and tissues, and their functions can vary depending on the specific annexin isoform and the cellular context. Annexins play important roles in cellular physiology, being involved in various processes, including membrane trafficking, signal transduction, apoptosis, inflammation, and ion channel regulation. A well‐known function of annexins is also their role in the maintenance of membrane integrity [48, 49, 69, 70].

Most members of the annexin family bind to the calcium‐binding S100 proteins [71, 72]. The first structure of a complex between an annexin and an S100 protein was solved in 1999 [73]. The complex contains S100A10, the only S100 protein that does not bind calcium and binds an N‐terminal peptide of AnxA2 (residues 1–13). S100A10 exists as a tight dimer and binds two AnxA2 molecules. This association modifies the distinct functions of both proteins. In 2000, the structure of a complex between calcium‐bound S100A11 in interaction with residues 1–14 of AnxA1 was solved [74]. AnxA6 is known to interact with S100A1, S100A6, S100A11, and S100B. AnxA2 binds to S100A10, S100A4, and S100A6. Conversely, S100A6 binds to AnxA2, AnxA6, and AnxA11. Dicalcin binds to AnxA1, AnxA2, and AnxA5. The N‐terminus of S100 proteins is always involved, whereas this is not true for the C‐terminus.

A remarkable property of several annexins is the capacity to self‐organize in two‐dimensional arrays [19, 75, 76]. The best characterized member of the family in this respect is AnxA5, which is the smallest annexin, consisting only of the conserved membrane‐binding core domain. AnxA5 has a high affinity for phosphatidylserine, a phospholipid that is the major negatively charged phospholipid in eukaryotic cells [77] and is normally located in the inner leaflet of the plasma membrane. In apoptotic cells, phosphatidylserine becomes exposed on the surface of apoptotic cells [78, 79], where it can undergo damage or stress. AnxA5 is able to bind the damaged membrane and participate in the repair and maintenance of membrane integrity by forming a two‐dimensional array.

However, overall, we are still far from having a global and satisfactory view of the annexin functions, giving the impression that we have only reached the tip of the iceberg.

### 2.5 Zooming on AnxA11

AnxA11 has recently become a major focus in amyotrophic lateral sclerosis research, especially after genetic studies identified mutations in the ANXA11 gene [80]. This is an incurable neurodegenerative disease that affects upper and lower motor neurons, resulting in death from neuromuscular respiratory failure. Several different clinically related mutations in AnxA11 were identified in a screening of a large cohort of familial ALS patients [80, 81]. In 2017, mutations in AnxA11 were first identified in patients with familial ALS (fALS) and later found in some sporadic ALS (sALS) cases. These mutations tend to cluster in the N‐terminal low‐complexity domain, involved in phase separation and protein‐protein interactions. Other mutations have been reported in the C‐terminal annexin repeats that are responsible for phospholipid binding and calcium regulation. Notable mutations are p.D40G, p.R235Q, and p.G38R, associated with early‐onset ALS, often with aggressive progression. As in many other neurodegenerative conditions, AnxA11‐related ALS is associated with, and thought to be caused by, protein aggregation and misfolding [80].

AnxA11 is a 56 kDa protein whose much longer N‐terminus comprises ∼200 residues. The N‐terminus contains a low‐complexity sequence in which up to circa one third of the residues are prolines and mediates interactions with a number of other proteins, among which are two EF‐hand calcium‐binding proteins: the apoptosis‐linked gene‐2 protein (ALG‐2) and S100A6 (calcyclin) [82]. It is in this N‐terminus that ALS‐associated mutations, p.D40G and p.G38R, were observed [80]. On the basis of a weak sequence homology with AnxA1, it was suggested that the N‐terminus of AnxA11 could contain a putative helical motif around residues 38 to 59 that could dictate, by analogy with AnxA1, a similar calcium‐dependent regulation [80]. Synthetic peptides containing the region 38–59 were indeed demonstrated experimentally to have a strong helical propensity [83]. Both the AnxA11 p.G38R and p.D40G ALS‐related variants were proven experimentally to abolish binding to calcyclin and induce aggregation of the full‐length protein [80]. These observations suggest that binding to calcyclin is an important element to enhance the AnxA11 solubility. Accordingly, a recent in vitro study has demonstrated that the β‐rich amyloid fibrils formed by recombinant AnxA11 N‐terminus and its disease‐associated variants can be redissolved in the presence of calcyclin [84]. Disease‐associated mutations, such as R235Q, are observed in the C‐terminal core domain: a variant of AnxA11 R235Q overexpressed in mouse primary motor neurons and human embryonic kidney (HEK) 293 cells was shown to produce high–molecular weight insoluble species able to sequester wild‐type AnxA11 similar to those observed in ALS patients [80]. However, structural considerations suggest that the mechanism is in this case different: the mutation affects a charged residue, R235Q, that in the structure (6tu2) is buried and forms a salt bridge with Glu461, which directly points toward the arginine in the protein core. This means that the mutation does not abolish an important molecular partner but rather destabilizes the fold, increasing the tendency to aggregate.

Finally, in predicting the interactome of AnxA11, we also need to consider the interaction with RNA. One of the surprising results of the analysis suggested by the catRAPID predictions is the indication that the N‐terminal region of AnxA11 (but not of the only apparently similar N‐terminus of AnxA7) could bind RNA [61]. This is somewhat surprising because this region is, as the N‐terminus of AnxA7, proline‐rich, that is, a residue not traditionally expected to be involved in RNA binding. Additionally, the N‐terminus does not contain positive charges or known RNA‐binding motifs. Nevertheless, RNA binding could be not sequence specific and be related instead to the tendency to give liquid‐liquid phase separation of this region. A library of bona fide RNA‐binding peptides also surprisingly contains proline‐rich motifs that could resemble the sequence of AnxA11.

Taken together, this evidence suggests an important functional role of AnxA11 despite the protein having attracted so far relatively less interest than other members of the annexin family.

### 2.6 Testing the AlphaFold Performance on the Isolated Proteins

Before attempting the prediction of the AnxA11 complexes, we tested how well AlphaFold or other prediction programs would perform on the individual proteins. We have discussed elsewhere the structure prediction of isolated AnxA11 and compared the results obtained with AlphaFold and RosETTA [83]. The structure of the conserved C‐terminal domain yields excellent results with both methods: the average RMSD values between the AlphaFold and RosETTA models and the calcium‐loaded x‐ray structure of AnxA11 (6tu2) are 0.9 Å and 1.2 Å, respectively. In both predictions, the N‐terminus is intrinsically disordered according to preliminary NMR and SAXS data that have confirmed the easy prediction that the protein contains a flexible intrinsically disordered N‐terminus and a globular C‐terminal domain [83]. Nevertheless, the RosETTA predictions suggest that the N‐terminal domain is completely separated from the C‐terminus, with the whole structure being elongated. The AlphaFold predictions suggest instead that the disordered N‐terminus wraps around, in a very loose and expanded way, the C‐terminal core. SAXS data seem to support the second model more [83].

**FIGURE 6:**
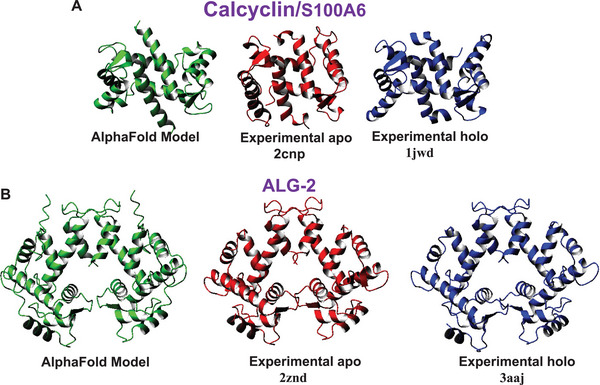
Modeling of the calcyclin and ALG‐2 dimers and comparison with the experimental structures of the apo and holo forms. The structures were first superimposed together and translated to allow comparison.

Both calcyclin and ALG‐2 belong to the calcium binding EF‐hand family and are known to be dimeric in solution [85, 86, 87]. Calcyclin is a member of the EF‐hand S100 subfamily and contains only one EF‐hand domain with two helix‐loop‐helix motifs connected by a flexible linker. The first putative calcium‐binding loop contains two extra residues, and calcium is coordinated only by carbonyls. The structures of both calcium‐free and calcium‐loaded calcyclin are known (2CNP and 1JWD, respectively), and they overlap on each other with an RMSD of 3.8 Å as a dimer and about the same when the individual monomers are considered. The AlphaFold models, obtained from https://alphafoldserver.com/, overlap fairly well with the experimental structure. However, the predictions are appreciably better for the calcium‐bound form than for the apo form, with RMSD of 3.0 Å and 4.3 Å, respectively (Figure 6A).

ALG‐2, the product of the PDCD6 gene, is also an EF‐hand protein with a flexible and hydrophobic Gly/Pro‐rich N‐terminal domain, and a C‐terminal calcium binding domain containing five helix‐loop‐helix motifs forming what is called a penta‐EF‐hand (PEF) domain. Reduced expression of ALG‐2 confers resistance to cell death induced by several stimuli, including glucocorticoids, T cell receptors, and Fas triggering. Thus, ALG‐2 was suggested to be a pro‐apoptotic protein that interacts with various target protein partners and functions as a Ca^2+^‐dependent adaptor in diverse cellular activities. The second EF‐hand is canonical with coordination through acidic residues. Monomer‐monomer association in the dimer occurs through the unpaired C‐terminal EF hand. The models predicted by AlphaFold superpose in the region 25–190 with an RMSD from the average of 0.3 Å as a monomer or 0.7 Å as dimers. The N‐terminus is disordered and thus was excluded in the modeling. The models fit the crystal structures of the apo and calcium‐loaded forms of dimeric ALG‐2 (2ZND and 3AAJ) with an RMSD of 0.80 Å and 1.5 Å, respectively, given that the apo and holo forms superpose with each other with an RMSD of 4.1 Å (Figure 6B). It is interesting to note that AlphaFold prefers one form of the structure over the other, with similar confidence.

### 2.7 The AnxA11 Complexes With EF‐Hand Proteins

**FIGURE 7:**
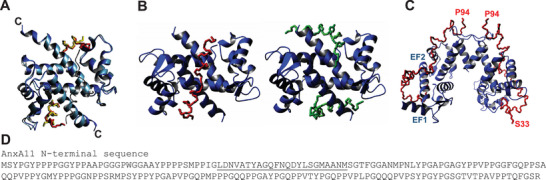
Modeling of the complexes between members of the annexin family and other proteins by AlphaFold (https://alphafoldserver.com/). (A) Model of the complex between S100A10 and the N‐terminus of AnxA2 (2:2 or 1:1 molar ratio) to understand the limits of the method. The ribbon model of S100A10 (dark blue) is superposed on the experimental structure, showing high accuracy (light blue, 1BT6). The experimental and modeled peptides from the AnxA2 N‐terminus are shown in yellow and red, respectively. (B) Ribbon model of S100A6 (blue) in complex with a peptide spanning the N‐terminus of AnxA11 (residues 39–61), assuming a 2:1 (left, red trace) and a 2:2 (right, green trace) stoichiometry for the interaction. (C) Model of the complex between dimeric ALG‐2 (blue) and an N‐terminal fragment of AnxA11 (red). (D) Sequence of the AnxA11 N‐terminus. The helical region is underlined. Along the sequences, there are several putative imperfect motifs that could potentially contribute to binding with the ALG‐2 protein.

We attempted to predict with AlphaFold the structure of the complexes of AnxA11 with calcyclin and ALG‐2. After preliminary runs using full‐length AnxA11, we decided to use only a peptide encompassing residues 37–60 because it is difficult to handle the long unstructured N‐terminal domain. This region has been indicated in several studies as the hotspot for the interaction [88]. To assess the degree of accuracy we could expect from the modeling, we first predicted the structure of the complex of AnxA2 with S100A10 that is already known (1BT6). This structure contains the S100A10 dimer bound to a peptide spanning residues 1 to 11 of AnxA2. The backbone atoms of residues 1–90 of the AlphaFold2 models of S100A10 overlap with the experimental structure with an RMSD of 1.4 Å. In the prediction, the AnxA2 peptides sit in practically identical positions as in the experimental structure, giving us further confidence in the potential accuracy of the modeling (Figure 7A).

We repeated the modeling for the complex between S100A6 and a peptide spanning residues 37–60 of AnxA11. The resulting models depend on the stoichiometry imposed. In both cases the peptide is helical, in agreement with experimental results [83], with a bend that makes it wrap around S100A6. However, if only one AnxA11 peptide is imposed, leading to a 2:1 S100A6:AnxA11 stoichiometry, the peptide helix is interrupted around residues 47–51 and sits across the two C‐terminal helices of S100A6, making contacts with both. If a 2:2 stoichiometry is assumed, as in all the structures of other annexins with S100 proteins, each peptide packs against the C‐terminal helix and the helix spanning residues 51–59 in a spatial position similar to that assumed by the N‐terminus of AnxA2 in the complex with S100A10. By analogy with other already known complexes with S100 members, we assume that the latter models are more reliable and should be considered the reference.

When we modeled the complex between the N‐terminus of AnxA11 (residues 33–63) and the structured domain of ALG‐2 assuming a 2:2 molar ratio, we obtained a model in which residues 39–47 and 51–66 again structured in two short helices that interact with helix 1 and 2 of EF1, and helix 1of EF1 and helix 4 of EF2, inserting into two grooves created between the two motifs (Figure 7B). Interestingly, EF1 is, with EF3, where calcium binds, inducing a small but clear conformational change that exposes more hydrophobic residues of ALG‐2. This would thus explain the calcium dependence of this interaction. Additional binding sites on the AnxA11 N‐terminus are possible and could comprise motifs similar to those involved in the interaction of ALG‐2 with the N‐terminus of AnxA11 that potentially contain several imperfect copies of the short peptide motifs named ABM‐1 (ALG‐2 binding motif type‐1) of PPYP(X)nYP (X, variable amino acids, *n* = 3–6) and ABM‐2 of [PΦ]PX[PΦ]G[FW]Ω ([PΦ], Pro or hydrophobic; [FW], Phe or Trp; Ω, large side chain; X, variable) that have been found to be needed for ALG‐2 recognition of the two protein partners ALG‐2 interacting protein X (ALIX) and the outer coat protein of coat protein complex II (COPII) Sec31A [89, 90].

While these models are, of course, still inherently inaccurate because many more tesserae are needed, they could nevertheless guide and inspire future studies to understand the mode of interaction between these proteins.

## 3 Conclusions

In this review, we have tried to provide an up‐to‐date perspective of the functions of annexins and discussed how this protein family can be involved in several quite different and yet potentially correlated cellular roles. We discussed in detail how the RNA‐binding properties known for some members of the family can be a more general and conserved feature of all annexins. We then focused on AnxA11 and discussed the interaction of this protein with EF‐hands. A better knowledge of the mechanisms of these interactions could be helpful both in understanding the role of AnxA11 in disease and in extending this knowledge to other annexins. Future directions will certainly require a more thorough cell biology effort to successfully link and integrate the different aspects of the annexin binding properties to their functions.

## Author Contributions

AP initiated the project, AV wrote the functional section, GGT wrote the RNA binding section. All three authors contributed to unify and finalize the text.

## Conflicts of Interest

The authors declare no conflicts of interest.

## References

[1] M. J. Geisow , J. H. Walker , C. Boustead , and W. Taylor , “Annexins–New Family of Ca2+‐Regulated‐Phospholipid Binding Protein,” Bioscience Reports 7, no. 4 (1987): 289–298. 2960386 10.1007/BF01121450

[2] M. J. Crumpton and J. R. Dedman , “Protein Terminology Tangle,” Nature 345, no. 6272 (1990): 212. 2333094 10.1038/345212a0

[3] J. Turnay , E. Lecona , S. Fernández‐Lizarbe , et al., “Structure‐function Relationship in Annexin A13, the Founder Member of the Vertebrate Family of Annexins,” Biochemical Journal 389, pt. 3 (2005): 899–911. 15813707 10.1042/BJ20041918 PMC1180741

[4] P. D. Smith and S. E. Moss , “Structural Evolution of the Annexin Supergene family,” Trends in Genetics 10, no. 7 (1994): 241–246. 8091504 10.1016/0168-9525(94)90171-6

[5] R. O. Morgan , D. W. Bell , J. R. Testa , and M. P. Fernandez , “Genomic Locations of ANX11 and ANX13 and the Evolutionary Genetics of Human Annexins,” Genomics 48, no. 1 (1998): 100–110. 9503022 10.1006/geno.1997.5148

[6] N. Quiskamp , M. Poeter , C. A. Raabe , et al., “The Tumor Suppressor Annexin A10 Is a Novel Component of Nuclear Paraspeckles,” Cellular and Molecular Life Sciences 71, no. 2 (2014): 311–329. 23715859 10.1007/s00018-013-1375-4 PMC11113197

[7] H. B. Ingram and A. H. Fox , “Unveiling the Intricacies of Paraspeckle Formation and Function,” Current Opinion in Cell Biology 90 (2024): 102399. 39033706 10.1016/j.ceb.2024.102399

[8] S. S. Patil , V. Panchal , T. Røstbø , et al., “RNA‐Binding Is an Ancient Trait of the Annexin Family,” Frontiers in Cell and Developmental Biology 11 (2023): 1161588. 37397259 10.3389/fcell.2023.1161588 PMC10311354

[9] A. Vedeler , H. Hollas , A. K. Grindheim , and A. M. Raddum , “Multiple Roles of Annexin A2 in Post‐Transcriptional Regulation of Gene Expression,” Current Protein & Peptide Science 13, no. 4 (2012): 401–412. 22708494 10.2174/138920312801619402

[10] Y. C. Liao , M. S. Fernandopulle , G. Wang , et al., “RNA Granules Hitchhike on Lysosomes for Long‐Distance Transport, Using Annexin A11 as a Molecular Tether,” Cell 179, no. 1 (2019): 147–164.e20. 31539493 10.1016/j.cell.2019.08.050 PMC6890474

[11] R. Huber , J. Römisch , and E. P. Paques , “The Crystal and Molecular Structure of Human Annexin V, an Anticoagulant Protein That Binds to Calcium and Membranes,” EMBO Journal 9, no. 12 (1990): 3867–3874. 2147412 10.1002/j.1460-2075.1990.tb07605.x PMC552154

[12] C. Thiel , K. Weber , and V. Gerke , “Characterization of a Ca(2+)‐Binding Site in Human Annexin II by Site‐Directed Mutagenesis,” Journal of Biological Chemistry 266 (1991): 14732–14739. 1830590

[13] H. Rosengarth AaL , “Annexin A2. Does It Induce Membrane Aggregation by a New Multimeric state of the Protein?” Annexins 1 (2004): 129–136.

[14] A. Rosengarth , J. Rösgen , H. J. Hinz , and V. Gerke , “Folding Energetics of Ligand Binding Proteins II. Cooperative Binding of Ca2+ to Annexin I,” Journal of Molecular Biology 306, no. 4 (2001): 825–835. 11243791 10.1006/jmbi.2000.4358

[15] A. Rosengarth and H. Luecke , “A Calcium‐Driven Conformational Switch of the N‐Terminal and Core Domains of Annexin A1,” Journal of Molecular Biology 326, no. 5 (2003): 1317–1325. 12595246 10.1016/s0022-2836(03)00027-5

[16] Y. H. Hong , H. S. Won , H. C. Ahn , and B. J. Lee , “Structural Elucidation of the Protein‐ and Membrane‐Binding Properties of the N‐Terminal Tail Domain of Human Annexin II,” Journal of Biochemistry 134, no. 3 (2003): 427–432. 14561728 10.1093/jb/mvg160

[17] I. Aukrust , H. Hollas , E. Strand , et al., “The mRNA‐Binding Site of Annexin A2 Resides in Helices C‐D of Its Domain IV,” Journal of Molecular Biology 368, no. 5 (2007): 1367–1378. 17395201 10.1016/j.jmb.2007.02.094

[18] E. Strand , H. Hollås , S. A. Sakya , et al., “Annexin A2 Binds the Internal Ribosomal Entry Site of c‐myc mRNA and Regulates Its Translation,” supplement, RNA Biology 18, no. S1 (2021): 337–354. 10.1080/15476286.2021.1947648 PMC8677036 34346292

[19] V. Gerke , C. E. Creutz , and S. E. Moss , “Annexins: Linking Ca2+ Signalling to Membrane Dynamics,” Nature Reviews Molecular Cell Biology 6, no. 6 (2005): 449–461. 15928709 10.1038/nrm1661

[20] V. Gerke , F. N. E. Gavins , M. Geisow , et al., “Annexins‐a Family of Proteins With Distinctive Tastes for Cell Signaling and Membrane Dynamics,” Nature Communications 15, no. 1 (2024): 1574. 10.1038/s41467-024-45954-0 PMC10882027 38383560

[21] V. Gerke and S. E. Moss , “Annexins: From Structure to Function,” Physiological Reviews 82, no. 2 (2002): 331–371. 11917092 10.1152/physrev.00030.2001

[22] A. L. Upton and S. E. Moss , “Molecular Cloning of a Novel N‐Terminal Variant of Annexin II from Rat Basophilic Leukaemia Cells,” Biochemical Journal 302, pt 2 (1994): 425–428. 8092993 10.1042/bj3020425 PMC1137245

[23] A. R. Nazmi , G. Ozorowski , M. Pejic , J. P. Whitelegge , V. Gerke , and H. Luecke , “N‐Terminal Acetylation of Annexin A2 Is Required for S100A10 Binding,” Biological Chemistry 393, no. 10 (2012): 1141–1150. 23091277 10.1515/hsz-2012-0179

[24] A. K. Grindheim , J. Saraste , and A. Vedeler , “Protein Phosphorylation and Its Role in the Regulation of Annexin A2 Function,” Biochimica Et Biophysica Acta ‐ General Subjects 1861, no. 11 pt. A (2017): 2515–2529. 28867585 10.1016/j.bbagen.2017.08.024

[25] S. U. Lauvrak , H. Hollas , A. P. Doskeland , I. Aukrust , T. Flatmark , and A. Vedeler , “Ubiquitinated Annexin A2 Is Enriched in the Cytoskeleton Fraction,” FEBS Letters 579, no. 1 (2005): 203–206. 15620714 10.1016/j.febslet.2004.11.076

[26] D. Caron , M. Boutchueng‐Djidjou , R. M. Tanguay , and R. L. Faure , “Annexin A2 Is SUMOylated on Its N‐terminal Domain: Regulation by Insulin,” FEBS Letters 589, no. 9 (2015): 985–991. 25775977 10.1016/j.febslet.2015.03.007

[27] M. V. Dorovkov , A. S. Kostyukova , and A. G. Ryazanov , “Phosphorylation of Annexin A1 by TRPM7 Kinase: A Switch Regulating the Induction of an α‐Helix,” Biochemistry 50, no. 12 (2011): 2187–2193. 21280599 10.1021/bi101963h PMC3062375

[28] M. Jost and V. Gerke , “Mapping of a Regulatory Important Site for Protein Kinase C Phosphorylation in the N‐Terminal Domain of Annexin II,” Biochimica Et Biophysica Acta 1313, no. 3 (1996): 283–289. 8898866 10.1016/0167-4889(96)00101-2

[29] S. E. Moss and R. O. Morgan , “The Annexins,” Genome Biology 5 (2004): 219. 15059252 10.1186/gb-2004-5-4-219 PMC395778

[30] U. Rescher and V. Gerke , “Annexins–Unique Membrane Binding Proteins With Diverse Functions,” Journal of Cell Science 117, pt. 13 (2004): 2631–2639. 15169834 10.1242/jcs.01245

[31] A. Bharadwaj , M. Bydoun , R. Holloway , and D. Waisman , “Annexin A2 Heterotetramer: Structure and Function,” International Journal of Molecular Sciences 14, no. 3 (2013): 6259–6305. 23519104 10.3390/ijms14036259 PMC3634455

[32] E. Aareskjold , A. K. Grindheim , H. Hollas , M. Goris , J. R. Lillehaug , and A. Vedeler , “Two Tales of Annexin A2 Knock‐Down: One of Compensatory Effects by Antisense RNA and Another of a Highly Active Hairpin Ribozyme,” Biochemical Pharmacology 166 (2019): 253–263. 31158338 10.1016/j.bcp.2019.05.028

[33] Y. Huang , H. Wang , and Y. Yang , “Annexin A7 Is Correlated With Better Clinical Outcomes of Patients With Breast Cancer,” Journal of Cellular Biochemistry 119 (2018): 7577–7584. 29893423 10.1002/jcb.27087

[34] L. D. Gibbs , K. Mansheim , S. Maji , et al., “Clinical Significance of Annexin A2 Expression in Breast Cancer Patients,” Cancers (Basel) 13, no. 1 (2020): 2. 33374917 10.3390/cancers13010002 PMC7792619

[35] A. P. Arrigo , J. L. Darlix , and P. F. Spahr , “A Cellular Protein Phosphorylated by the Avian Sarcoma Virus Transforming Gene Product Is Associated With Ribonucleoprotein Particles,” EMBO Journal 2, no. 3 (1983): 309–315. 11894943 10.1002/j.1460-2075.1983.tb01424.x PMC555134

[36] A. Vedeler and H. Hollas , “Annexin II Is Associated With mRNAs Which May Constitute a Distinct Subpopulation,” Biochemical Journal 348, pt. 3 (2000): 565–572. 10839987 PMC1221098

[37] K. Monastyrskaya , “Functional Association Between Regulatory RNAs and the Annexins,” International Journal of Molecular Sciences 19, no. 2 (2018): 591. 29462943 10.3390/ijms19020591 PMC5855813

[38] I. Mickleburgh , B. Burtle , H. Hollas , et al., “Annexin A2 Binds to the Localization Signal in the 3' Untranslated Region of c‐myc mRNA,” FEBS Journal 272 (2005): 413–421. 15654879 10.1111/j.1742-4658.2004.04481.x

[39] H. Hollas , I. Aukrust , S. Grimmer , E. Strand , T. Flatmark , and A. Vedeler , “Annexin A2 Recognises a Specific Region in the 3'‐UTR of Its Cognate Messenger RNA,” Biochimica Et Biophysica Acta 1763, no. 11 (2006): 1325–1334. 17045350 10.1016/j.bbamcr.2006.08.043

[40] N. R. Filipenko , T. J. MacLeod , C. S. Yoon , and D. M. Waisman , “Annexin A2 Is a Novel RNA‐Binding Protein,” Journal of Biological Chemistry 279, no. 10 (2004): 8723–8731. 14672933 10.1074/jbc.M311951200

[41] A. Anji and M. Kumari , “A Cis‐acting Region in the N‐Methyl‐d‐Aspartate R1 3'‐Untranslated Region Interacts With the Novel RNA‐Binding Proteins Beta Subunit of Alpha Glucosidase II and Annexin A2–Effect of Chronic Ethanol Exposure In Vivo,” European Journal of Neuroscience 34, no. 8 (2011): 1200–1211. 21995826 10.1111/j.1460-9568.2011.07857.x PMC3195980

[42] A. Hirata and F. Hirata , “Lipocortin (Annexin) I Heterotetramer Binds to Purine RNA and Pyrimidine DNA,” Biochemical and Biophysical Research Communications 265 (1999): 200–204. 10548514 10.1006/bbrc.1999.1660

[43] D. K. Nabariya , A. Heinz , S. Derksen , and S. Krauß , “Intracellular and Intercellular Transport of RNA Organelles in CXG Repeat Disorders: The Strength of Weak Ties,” Frontiers in Molecular Biosciences 9 (2022): 1000932. 36589236 10.3389/fmolb.2022.1000932 PMC9800848

[44] M. K. Vorländer , B. Pacheco‐Fiallos , and C. Plaschka , “Structural Basis of mRNA Maturation: Time to Put It Together,” Current Opinion in Structural Biology 75 (2022): 102431. 35930970 10.1016/j.sbi.2022.102431

[45] I. Aukrust , L. A. Rosenberg , M. M. Ankerud , et al., “Post‐translational Modifications of Annexin A2 Are Linked to Its Association With Perinuclear Nonpolysomal mRNP Complexes,” FEBS Open Bio 7, no. 2 (2017): 160–173. 10.1002/2211-5463.12173 PMC5292671 28174683

[46] A. Khong and R. Parker , “The Landscape of Eukaryotic mRNPs,” RNA (New York, NY) 26 (2020): 229–239. 10.1261/rna.073601.119 PMC7025503 31879280

[47] N. Kedersha , G. Stoecklin , M. Ayodele , et al., “Stress Granules and Processing Bodies Are Dynamically Linked Sites of mRNP Remodeling,” Journal of Cell Biology 169, no. 6 (2005): 871–884. 15967811 10.1083/jcb.200502088 PMC2171635

[48] T. L. Boye and J. Nylandsted , “Annexins in Plasma Membrane Repair,” Biological Chemistry 397 (2016): 961–969. 27341560 10.1515/hsz-2016-0171

[49] S. N. Koerdt , A. P. K. Ashraf , and V. Gerke , “Annexins and Plasma Membrane Repair,” Current Topics in Membranes 84 (2019): 43–65. 31610865 10.1016/bs.ctm.2019.07.006

[50] J. Ayala‐Sanmartin , M. Vincent , J. Sopkova , and J. Gallay , “Modulation by Ca(2+) and by Membrane Binding of the Dynamics of Domain III of Annexin 2 (p36) and the Annexin 2‐p11 Complex (p90): Implications for Their Biochemical Properties,” Biochemistry 39, no. 49 (2000): 15179–15189. 11106497 10.1021/bi000501x

[51] A. K. Grindheim , S. S. Patil , C. G. Nebigil , L. Désaubry , and A. Vedeler , “The Flavagline FL3 Interferes With the Association of Annexin A2 With the eIF4F Initiation Complex and Transiently Stimulates the Translation of Annexin A2 mRNA,” Frontiers in Cell and Developmental Biology 11 (2023): 1094941. 37250892 10.3389/fcell.2023.1094941 PMC10214161

[52] C. V. Nicchitta , R. S. Lerner , S. B. Stephens , R. D. Dodd , and B. Pyhtila , “Pathways for Compartmentalizing Protein Synthesis in Eukaryotic Cells: The Template‐Partitioning Model,” Biochemistry and Cell Biology 83 (2005): 687–695. 16333319 10.1139/o05-147

[53] W. C. Merrick and G. D. Pavitt , “Protein Synthesis Initiation in Eukaryotic Cells,” Cold Spring Harbor Perspectives in Biology 10, no. 12 (2018): a033092. 29735639 10.1101/cshperspect.a033092 PMC6280705

[54] T. Kwan and S. R. Thompson , “Noncanonical Translation Initiation in Eukaryotes,” Cold Spring Harbor Perspectives in Biology 11, no. 4 (2019): a032672. 29959190 10.1101/cshperspect.a032672 PMC6442200

[55] L. C. Cobbold , K. A. Spriggs , S. J. Haines , et al., “Identification of Internal Ribosome Entry Segment (IRES)‐Trans‐Acting Factors for the Myc Family of IRESs,” Molecular and Cellular Biology 28 (2008): 40–49. 17967896 10.1128/MCB.01298-07 PMC2223313

[56] I.‐W. Park , H. K. Fiadjoe , and P. Chaudhary , “Impact of Annexin A2 on Virus Life Cycles,” Virus Research 345 (2024): 199384. 38702018 10.1016/j.virusres.2024.199384 PMC11091703

[57] E. V. Ryzhova , R. M. Vos , A. V. Albright , A. V. Harrist , T. Harvey , and F. González‐Scarano , “Annexin 2: A Novel Human Immunodeficiency Virus Type 1 Gag Binding Protein Involved in Replication in Monocyte‐Derived Macrophages,” Journal of Virology 80, no. 6 (2006): 2694–2704. 16501079 10.1128/JVI.80.6.2694-2704.2006 PMC1395445

[58] S. M. O. Solbak , E. Abdurakhmanov , A. Vedeler , and U. H. Danielson , “Characterization of Interactions Between Hepatitis C Virus NS5B Polymerase, Annexin A2 and RNA—Effects on NS5B Catalysis and Allosteric Inhibition,” Virology Journal 14, no. 1 (2017): 236. 29228983 10.1186/s12985-017-0904-4 PMC5725786

[59] V. Saxena , C. K. Lai , T. C. Chao , K. S. Jeng , and M. M. Lai , “Annexin A2 Is Involved in the Formation of Hepatitis C Virus Replication Complex on the Lipid Raft,” Journal of Virology 86 (2012): 4139–4150. 22301157 10.1128/JVI.06327-11 PMC3318618

[60] H. Kwak , M. W. Park , and S. Jeong , “Annexin A2 Binds RNA and Reduces the Frameshifting Efficiency of Infectious Bronchitis Virus,” PLoS ONE 6, no. 8 (2011): 24067. 10.1371/journal.pone.0024067 PMC3168876 21918681

[61] G. G. Tartaglia , H. Hollås , B. Håvik , A. Vedeler , and A. Pastore , “The RNA‐Binding Properties of Annexins,” Journal of Molecular Biology 437 (2025): 168933. 39755246 10.1016/j.jmb.2024.168933

[62] M. Bellucci , F. Agostini , M. Masin , and G. G. Tartaglia , “Predicting Protein Associations With Long Noncoding RNAs,” Nature Methods 8, no. 6 (2011): 444–445. 21623348 10.1038/nmeth.1611

[63] A. Armaos , A. Colantoni , G. Proietti , J. Rupert , and G. G. Tartaglia , “catRAPID Omics v2.0: Going Deeper and Wider in the Prediction of Protein‐RNA Interactions,” Nucleic Acids Research 49 (2021): W72–W79. 34086933 10.1093/nar/gkab393 PMC8262727

[64] X. H. Xu , W. Pan , L. H. Kang , H. Feng , and Y. Q. Song , “Association of Annexin A2 With Cancer Development (Review),” Oncology Reports 33, no. 5 (2015): 2121–2128. 25760910 10.3892/or.2015.3837

[65] K. Gao , X. Li , S. Luo , and L. Zhao , “An Overview of the Regulatory Role of Annexin A1 in the Tumor Microenvironment and Its Prospective Clinical Application (Review),” International Journal of Oncology 64 (2024): 51. 38516766 10.3892/ijo.2024.5639 PMC10997369

[66] J. Cao , S. Wan , S. Chen , and L. Yang , “ANXA6: A Key Molecular Player in Cancer Progression and Drug Resistance,” Discover Oncology 14, no. 1 (2023): 53. 37129645 10.1007/s12672-023-00662-x PMC10154440

[67] H. Zhang , Z. Zhang , T. Guo , et al., “Annexin A Protein Family: Focusing on the Occurrence, Progression and Treatment of Cancer,” Frontiers in Cell and Developmental Biology 11 (2023): 1141331. 36936694 10.3389/fcell.2023.1141331 PMC10020606

[68] A. Naskar , A. Nayak , M. R. Salaikumaran , S. S. Vishal , and P. P. Gopal , “Phase Separation and Pathologic Transitions of RNP Condensates in Neurons: Implications for Amyotrophic Lateral Sclerosis, Frontotemporal Dementia and Other Neurodegenerative Disorders,” Frontiers in Molecular Neuroscience 16 (2023): 1242925. 37720552 10.3389/fnmol.2023.1242925 PMC10502346

[69] C. Croissant , R. Carmeille , C. Brévart , and A. Bouter , “Annexins and Membrane Repair Dysfunctions in Muscular Dystrophies,” International Journal of Molecular Sciences 22, no. 10 (2021): 5276. 34067866 10.3390/ijms22105276 PMC8155887

[70] A. Draeger , K. Monastyrskaya , and E. B. Babiychuk , “Plasma Membrane Repair and Cellular Damage Control: The Annexin Survival Kit,” Biochemical Pharmacology 81, no. 6 (2011): 703–712. 21219882 10.1016/j.bcp.2010.12.027

[71] J. Weisz and V. N. Uversky , “Zooming Into the Dark Side of Human Annexin‐S100 Complexes: Dynamic Alliance of Flexible Partners,” International Journal of Molecular Sciences 21, no. 16 (2020): 5879. 32824294 10.3390/ijms21165879 PMC7461550

[72] P. Ecsédi , G. Gógl , and L. Nyitray , “Studying the Structures of Relaxed and Fuzzy Interactions: The Diverse World of S100 Complexes,” Frontiers in Molecular Biosciences 8 (2021): 749052. 34708078 10.3389/fmolb.2021.749052 PMC8542695

[73] S. Réty , J. Sopkova , M. Renouard , et al., “The Crystal Structure of a Complex of p11 With the Annexin II N‐Terminal Peptide,” Natural Structural Biology 6, no. 1 (1999): 89–95. 10.1038/4965 9886297

[74] S. Réty , D. Osterloh , J. P. Arié , et al., “Structural Basis of the Ca(2+)‐Dependent Association between S100C (S100A11) and Its Target, the N‐Terminal Part of Annexin I,” Structure (London, England: 1993) 8 (2000): 175–184. 10673436 10.1016/s0969-2126(00)00093-9

[75] R. P. Richter , J. L. Him , B. Tessier , C. Tessier , and A. R. Brisson , “On the Kinetics of Adsorption and Two‐Dimensional Self‐Assembly of Annexin A5 on Supported Lipid Bilayers,” Biophysical Journal 89 (2005): 3372–3385. 16085777 10.1529/biophysj.105.064337 PMC1366834

[76] G. Mosser , C. Ravanat , J. M. Freyssinet , and A. Brisson , “Sub‐domain Structure of Lipid‐Bound Annexin‐V Resolved by Electron Image Analysis,” Journal of Molecular Biology 217, no. 2 (1991): 241–245. 1825119 10.1016/0022-2836(91)90538-h

[77] P. F. Devaux , “Protein Involvement in Transmembrane Lipid Asymmetry,” Annual Review of Biophysics and Biomolecular Structure 21 (1992): 417–439. 10.1146/annurev.bb.21.060192.002221 1525472

[78] A. Bouter , C. Gounou , R. Bérat , et al., “Annexin‐A5 Assembled Into Two‐Dimensional Arrays Promotes Cell Membrane Repair,” Nature Communications 2 (2011): 270. 10.1038/ncomms1270 PMC3104517 21468022

[79] A. Bouter , R. Carmeille , C. Gounou , et al., “Review: Annexin‐A5 and Cell Membrane Repair,” supplement, Placenta 36, no. S1 (2015): S43–S49. 10.1016/j.placenta.2015.01.193 25701430

[80] B. N. Smith , S. D. Topp , C. Fallini , et al., “Mutations in the Vesicular Trafficking Protein Annexin A11 Are Associated With Amyotrophic Lateral Sclerosis,” Science Translational Medicine 9, no. 388 (2017): aad9157. 10.1126/scitranslmed.aad9157 PMC6599403 28469040

[81] E. Teyssou , F. Muratet , M. D. Amador , et al., “Genetic Screening of ANXA11 Revealed Novel Mutations Linked to Amyotrophic Lateral Sclerosis,” Neurobiology of Aging 99 (2021): 102.e11–102.e20. 10.1016/j.neurobiolaging.2020.10.015 33218681

[82] A. C. Rintala‐Dempsey , A. Rezvanpour , and G. S. Shaw , “S100‐Annexin Complexes–Structural Insights,” FEBS Journal 275 (2008): 4956–4966. 18795951 10.1111/j.1742-4658.2008.06654.x

[83] E. F. Dudas , M. D. Tully , T. Foldes , et al., “The Structural Properties of Full‐length Annexin A11,” Frontiers in Molecular Biosciences 11 (2024): 1347741. 38516187 10.3389/fmolb.2024.1347741 PMC10955470

[84] A. Shihora , R. D. Elias , J. A. Hammond , et al., “ALS Variants of Annexin A11's Proline‐Rich Domain Impair Its S100A6‐Mediated Fibril Dissolution,” ACS Chemical Neuroscience 14, no. 15 (2023): 2583–2589. 37433222 10.1021/acschemneuro.3c00169 PMC10401653

[85] J. Elíes , M. Yáñez , T. M. C. Pereira , J. Gil‐Longo , D. A. MacDougall , and M. Campos‐Toimil , “An Update to Calcium Binding Proteins,” Advances in Experimental Medicine and Biology 1131 (2020): 183–213. 31646511 10.1007/978-3-030-12457-1_8

[86] T. Sudo and H. Hidaka , “Regulation of Calcyclin (S100A6) Binding by Alternative Splicing in the N‐Terminal Regulatory Domain of Annexin XI Isoforms,” Journal of Biological Chemistry 273, no. 11 (1998): 6351–6357. 9497364 10.1074/jbc.273.11.6351

[87] H. Satoh , Y. Nakano , H. Shibata , and M. Maki , “The Penta‐EF‐Hand Domain of ALG‐2 Interacts With Amino‐Terminal Domains of Both Annexin VII and Annexin XI in a Ca2+‐Dependent Manner,” Biochimica Et Biophysica Acta 1600 (2002): 61–67. 12445460 10.1016/s1570-9639(02)00445-4

[88] J. Wang , C. Guo , S. Liu , et al., “Annexin A11 in Disease,” Clinica Chimica Acta 431 (2014): 164–168. 10.1016/j.cca.2014.01.031 24508622

[89] H. Suzuki , M. Kawasaki , T. Inuzuka , et al., “Structural Basis for Ca2+ ‐Dependent Formation of ALG‐2/Alix Peptide Complex: Ca2+/EF3‐Driven Arginine Switch Mechanism,” Structure (London, England: 1993) 16 (2008): 1562–1573. 18940611 10.1016/j.str.2008.07.012

[90] T. Takahashi , K. Kojima , W. Zhang , et al., “Structural Analysis of the Complex between Penta‐EF‐Hand ALG‐2 Protein and Sec31A Peptide Reveals a Novel Target Recognition Mechanism of ALG‐2,” International Journal of Molecular Sciences 16, no. 2 (2015): 3677–3699. 25667979 10.3390/ijms16023677 PMC4346919

